# Investigating the potential of the anti-epileptic drug imepitoin as a treatment for co-morbid anxiety in dogs with idiopathic epilepsy

**DOI:** 10.1186/s12917-017-1000-0

**Published:** 2017-04-07

**Authors:** Rowena M. A. Packer, Luisa De Risio, Holger A. Volk

**Affiliations:** 1 Department of Clinical Science and Services, Royal Veterinary College, Hatfield, Hertfordshire AL9 7TA UK; 2 Animal Health Trust, Newmarket, Suffolk CB8 7UU UK

**Keywords:** Canine, Epilepsy, Anxiolytic, Anxiety, Fear, Behaviour, Seizure, Idiopathic

## Abstract

**Background:**

Behavioural changes associated with idiopathic epilepsy (IE) have been identified in dogs, with fear and anxiety-related problems seen in both drug-naïve dogs and dogs treated with anti-epileptic drugs (AEDs). Treating anxiety-related behaviour in dogs with IE may be challenging, as seizures are a contraindication for many conventional anxiolytic drugs. In addition, many dogs with IE are already treated with AEDs to reduce their seizure frequency, which may have negative effects if used in polytherapy. Imepitoin is low-affinity partial agonist at the benzodiazepine (BDZ) site of the GABA_A_ receptor, and has been demonstrated to have both anticonvulsant and anxiolytic effects in laboratory rodents. Imepitoin has been developed for the treatment of IE in dogs, with demonstrated anticonvulsant effects and high tolerability and safety. To date, imepitoin’s potential to reduce anxiety in dogs with IE has not been investigated. An online survey was conducted to investigate the effect of imepitoin on fear and anxiety-related behaviours in dogs with IE. Eighty-five valid responses were received from owners of dogs with IE currently treated with imepitoin. Anxiety-related behaviour was quantified before and during imepitoin treatment using a validated questionnaire tool (C-BARQ).

**Results:**

No differences were observed in the five fear/anxiety-related measures between the two time periods (before vs. during treatment) for dog directed fear, stranger directed fear, non-social fear, pain sensitivity and separation related behaviour. A median 45% reduction in seizure frequency/month was observed following imepitoin treatment; however, imepitoin did not appear effective in reducing seizure frequency in a minority of cases. Polyphagia was the most common chronic side effect, and more side effects were reported in polytherapy cases.

**Conclusions:**

Imepitoin does not appear to improve anxiety-related behaviour in dogs with IE treated with this medication for its anti-epileptic effects. Investigating the effects of imepitoin upon the behaviour of dogs with recognised behavioural anxiety-related problems (e.g. specific fears and phobias, separation related behaviours), in both healthy dogs and dogs with epilepsy is required to further explore any potential anxiolytic effects of this medication.

## Background

There is mounting evidence that epilepsy is not simply a seizure disorder, with the prevalence of psychiatric disorders in people with epilepsy higher than in either the general population or patients with other chronic medical diseases [1–4]. The most common co-morbid psychiatric disorders of epilepsy in people are anxiety and depression [2, 5–8]. Comorbidities may be a cause of epilepsy, a consequence of epilepsy, or a separate condition that is associated with epilepsy because there is a common cause for the epilepsy and the comorbidity [9]. The impact of co-morbid psychiatric disorders upon patient quality of life (QoL) may be severe, with inter-ictal anxiety and depression found to have greater adverse effects on QoL than those of seizure frequency, severity and chronicity [10]. As such, treatment that does not simply focus upon seizure control may be beneficial to the QoL of patients with epilepsy [9].

Co-morbid anxiety has recently been recognised in dogs with idiopathic epilepsy (IE) [11], with behavioural changes including increases in fear and anxiety associated with the development of IE [11]. Increased fear and anxiety were observed in drug-naïve dogs as well as those treated with AEDs, indicating these behavioural changes were not merely a treatment side effect [11]. For example, in a recent study of epilepsy in the Italian Spinone, 55% of dogs developed behavioural abnormalities (commonly associated with anxiety) at epilepsy onset, before the initiation of AEDs [12]. Due to the potentially severe impact of anxiety upon QoL, addressing this behavioural change alongside managing seizures is desirable.

There are few psychopharmacological agents licensed for use in dogs, and treating anxiety-related behaviour in dogs with IE may be additionally challenging, as epilepsy or a history of seizures are a contraindication for many anxiolytic drugs. This includes the tri-cyclic antidepressant (TCA) clomipramine, buspirone, and the selective serotonin reuptake inhibitors (SSRI) fluoxetine, imipramine, paroxetine, sertraline, amitriptyline and fluvoxamine [13]. As such, there is a need for anxiolytic drugs that:

(i) Reduce anxiety without reducing the seizure threshold in dogs with IE;
(ii) Have the potential to be administered as a polytherapy alongside existing AEDs; and
(iii) Have a favourable side effect profile to allow their long-term administration, if required.

Imepitoin is low-affinity partial agonist at the benzodiazepine (BDZ) binding site of the GABA_A_ receptor. Partial activation of the GABA receptors by imepitoin has been demonstrated to have pronounced anti-seizure activity and anxiolytic activity in rodent models, with a high tolerability [14]. The anxiolytic effects of imepitoin have been demonstrated in the rat in three experimental paradigms, the Vogel conflict test, the elevated plus maze and the light-dark box [15]. Imepitoin has been developed as an antiepileptic drug for canine idiopathic epilepsy (IE), with multiple studies indicating that it is well tolerated in dogs, with antiepileptic effects observed in both newly diagnosed IE cases as monotherapy and in drug-resistant dogs as add-on treatment to phenobarbital [16–20]. The anxiolytic effects of imepitoin have yet to be demonstrated in detail in the dog; however, anecdotal evidence from the first clinical studies of imepitoin indicated that owners perceived that their dog’s epilepsy-related behavioural changes had improved during imepitoin treatment, an effect not previously seen with other anti-epileptic drugs (AEDs) such as phenobarbital [21]. Imepitoin has potential benefits over existing anxiolytics with a fast onset of action ~2–3 h post oral dosing [14, 22], compared to around 3–5 weeks for SSRIs and TCAs [23]. To date, the only behavioural effect of imepitoin that has been reported is hyperactivity [18], which may be expected as paradoxical excitement can occur in dogs treated with benzodiazepines (e.g. diazepam) [24].

Whether imepitoin, a BDZ partial agonist, can be both an effective anticonvulsant and anxiolytic in dogs with IE requires further investigation. The purpose of this study was to investigate the effect of imepitoin upon owner-reported indicators of fear and anxiety-related behaviour in dogs treated with imepitoin for IE, to investigate imepitoin’s impact when administered as a polytherapy alongside other AEDs, and to quantify imepitoin’s side effect profile when used as a mono- or polytherapy.

## Methods

### Recruitment

A web-based questionnaire study was conducted to investigate behavioural effects of imepitoin in dogs treated with this medication for IE. Owners of dogs currently being treated with imepitoin for IE were recruited online via online (canine epilepsy forums, Twitter, Facebook pages, the Royal Veterinary College and Animal Health Trust websites) and via posters and leaflets at local veterinary practices and breed association groups. To be included in the study, dogs must have:

1. Been diagnosed with IE either:

i. By a first opinion vet due to recurrent seizures for more than 1 year, with the first seizure having occurred while aged between 6 months to 6 years, with no identifiable cause found (Tier I confidence level for the diagnosis of IE [25]);﻿ OR
ii. By a specialist in veterinary neurology with the aid of blood tests (minimum data base blood tests [25]) and MRI of the brain

(2) Been treated with imepitoin for IE for a minimum of 4 weeks (28 days).

All dogs currently being treated with imepitoin were eligible for this study, regardless of AED history to include a breadth of cases. No pre-screening of anxiety levels was conducted prior to inclusion in the study, to allow the investigation of imepitoin’s effects upon dogs with a wide spectrum of anxiety levels. Consent was gained via a statement at the start of the questionnaire, after the purpose of the study and potential use of the results had been explained to the owner.

### Questionnaire content

The survey was entitled “A questionnaire study of the impact of Pexion (Imepitoin) on canine behaviour and welfare” as not to bias responses towards owners of dogs with anxiety problems, or dogs who had experienced marked changes in anxiety following imepitoin treatment. The survey was structured into four sections: clinical history, behavioural changes, treatment efficacy and side effects profile.

#### Clinical history

Owners reported their dog’s signalment, pedigree, age at first seizure, seizure history, diagnostic tests performed to diagnose IE, seizure frequency, experience of cluster seizures, and frequency of cluster seizures and status epilepticus (as defined by [26]), seizure semiology and seizure recovery characteristics. AED treatment history including the use of other anti-epileptic drugs was recorded, and whether the dog was currently being treated with imepitoin as a monotherapy or polytherapy. Owners were asked to report their dog’s current dose of imepitoin (mg) and their dog’s weight (kg).

#### Behavioural changes

Behavioural traits were quantified using a validated questionnaire tool, the Canine Behavioral Assessment and Research Questionnaire (C-BARQ) [27]. Owners were questioned on their dog’s anxiety-related behaviour before and during imepitoin treatment using five of the eleven C-BARQ behavioural measures: ‘Dog directed fear’, ‘Stranger directed fear’; ‘Non-social fear’; ‘Pain sensitivity’ and ‘Separation related behaviour’. The C-BARQ has previously been used to quantify neurobehavioral changes in dogs with neurological disease, including IE and syringomyelia [11, 28] and to quantify the impact of a ketogenic dietary therapy upon the behaviour of dogs with IE in a placebo-controlled crossover trial [29].

#### Treatment efficacy

The effect of imepitoin on seizure activity was recorded via owner reports of the following measures both *before* and *during* imepitoin treatment:

(i) Mean number of seizures/month;
(ii) Mean number of seizure days/month;
(iii) Mean number of cluster seizures/month (how many times their dog experienced more than one seizure in a 24 h period per month).

Owners were asked to estimate these measures from the 3 months prior to imepitoin use, and for the entire length of time they had been treated with imepitoin for. The percentage change in the number of seizures/month was calculated from measure (i) compared before and during imepitoin treatment.

#### Side effects profile

Owners were questioned on the presence and severity of 11 AED side effects in the first 2 weeks of imepitoin treatment, and after 2 weeks of imepitoin treatment: polyphagia, polydipsia, polyuria, weight gain, increased sleeping, ataxia, restlessness, pruritus, vomiting, diarrhoea and coughing. Owners were asked to report the severity of these side effects on a five point scale of very mild (1), mild (2), moderate (3), severe (4), and very severe (5). Composite side effect scores were compiled for the initial 2 weeks of treatment and after 2 weeks of treatment by adding the scores of each of the 11 signs (out of 55).

### Statistical analysis

The five C-BARQ measures were calculated for each dog using weighted scores, calculated using the loading factors for each question established in the original C-BARQ validation study multiplied by the owner’s response to each question. Descriptive statistics were calculated for each behavioural measure. Effects of treatment protocol (dogs receiving mono or polytherapy with imepitoin), cluster seizures, signalment and seizure frequency before treatment were investigated for each behavioural trait, measures of drug response and side effect profiles. Data were visually examined for normality of distribution using histograms. The Wilcoxon test was used to compare continuous paired data e.g. C-BARQ measures before and during imepitoin use, the Mann-Whitey (U) tests for comparing continuous independent data between two groups, and the Kruskall-Wallis (KW) test for comparing continuous independent data between more than two groups. Chi-squared (*X* ^2^) analysis was used to investigate associations between categorical variables. All tests were two-sided and *P* < 0.05 was considered to be significant. Data are presented as mean (±SD), or median (25th–75th quartile), where appropriate.

## Results

### Responses

Responses were received from 176 owners of which 85 were valid responses. Responses were deemed invalid (*n* = 91) for the following reasons: *n* = 74 were incomplete and could not be analysed, *n* = 9 dogs were deceased at the time of response, *n* = 4 dogs were not receiving imepitoin at the time of response, *n* = 2 dogs were over the limit of age at first seizure (without further veterinary diagnostics to increase the confidence of an IE diagnosis), and *n* = 2 had been diagnosed with structural epilepsy (caused by identified cerebral pathology) [26].

### Demographics – Owner

The majority of the owners responding were female (77%, *n* = 65), with the most common age group being 46–60 years old (47.6%, *n* = 40), followed by 31–45 (32.1%, *n* = 27). The majority of responses were from the UK (92.9%, *n* = 78), with 3.6% from Germany (*n* = 3), 1.2% (*n* = 1) from Belgium, Spain, Sweden and *n* = 1 owner did not disclose their location. The majority of owners had heard about the survey via social media (60.7%, *n* = 51), followed by web searches (21.4%, *n* = 18), their regular vet (7.1%, *n* = 6) and their specialist vet (6.0%, *n* = 5). Nearly one third of dogs had experienced a seizure within a week of their owner completing the survey (31.8%, *n* = 27), and a further 28.2% 1–3 weeks prior to completion.

### Demographics - dog

Nearly two thirds of dogs were male (male neutered: 55.3%, *n* = 47, male entire: 10.6%, *n* = 9), with around one third female (female neutered: 30.6%, *n* = 26, female entire 3.5%, *n* = 3). The mean age (months) ± SD was 57.7 ± 29.6 months and mean weight (kg) ± SD was 23.1 ± 12.3. The majority of dogs were pure bred (78.8%, *n* = 67), with 34 different breeds represented, and 21.2% were cross-bred (*n* = 18). Almost half of all dogs were Kennel Club registered (48.2%, *n* = 41). The most common breeds represented were the Labrador Retriever (12.9%, *n* = 11), Border Collie (10.6%, *n* = 9), Beagle (3.5%, *n* = 3), Golden Retriever (3.5%, *n* = 3) and Italian Spinone (3.5%, *n* = 3).

### Epilepsy phenotype

The median age (months) at first seizure (25th–75th percentile) was 28.5 (18–46.3) months. The median total number of seizures a dog had experienced was 12 (8–30). Over half of dogs (55.3%, *n* = 47) had experienced cluster seizures (more than one fit within 24 h), with 43.5% only experiencing single seizures (*n* = 37) and 1.2% (*n* = 1) of owners unsure.

### Anti-epileptic drug treatment

The median time in days from first seizure to imepitoin treatment was 275.0 (105.0–743.0). Imepitoin was started after 2–5 seizures in the majority of dogs (41.2%, *n* = 35), and after the first seizure in only 9.4% (*n* = 11) of dogs. The median duration of imepitoin treatment was 263.9 days (106.3–478.4). The mean dose of imepitoin was 23.37 mg/kg (SD: 6.43). The lowest dose was 10.0 mg/kg and the highest was 35.7 mg/kg, with the majority of dogs receiving a dose between 20 and 30 mg/kg (53.5%).

**Fig. 1:**
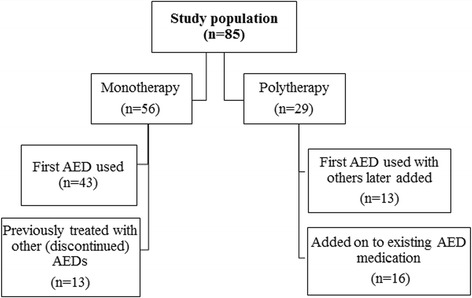
Different uses of imepitoin as a monotherapy or a polytherapy in a study population of 85 dogs treated with imepitoin for idiopathic epilepsy

Imepitoin was the first anti-epileptic medication received for 65.9% of dogs (*n* = 56), with the other third of dogs (32.9%, *n* = 28) having already received other anti-epileptic drugs (AEDs). The majority of these dogs had already been treated with phenobarbital (PB) (*n* = 22), followed by potassium bromide (KBr) (*n* = 8), and levetiracetam (LEV) (*n* = 3). At the time of the survey, two thirds of dogs were being treated with imepitoin as a monotherapy (65.9%, *n* = 56) and one third were being treated as a polytherapy (34.1%, *n* = 29) (Fig. 1). Of those dogs receiving polytherapy, *n* = 21 were being treated with PB, *n* = 13 with KBr and *n* = 8 with LEV. Nearly one quarter of owners used rectal diazepam as an emergency treatment (23.5%, *n* = 20).

Of the monotherapy cases (*n* = 56), imepitoin was the first AED they were treated with for 76.8% of cases (*n* = 43), with the remaining 21.4% (*n* = 13) having previously been treated with other AED (s) whose use had since been discontinued. Of the polytherapy cases (*n* = 29), 44.8% (*n* = 13) had received imepitoin as their dogs first AED with additional AEDs added at a later date, and 55.2% (*n* = 16) received imepitoin as an add-on to existing AED medication. There was a significant association between cluster seizures and therapy type, with dogs that only experienced single seizures more likely to be treated with imepitoin as a monotherapy (83.6%) than as a polytherapy (16.2%) (*X* ^2^ = 9.81, *p* = 0.002).

### Behavioural effects

**Fig. 2:**
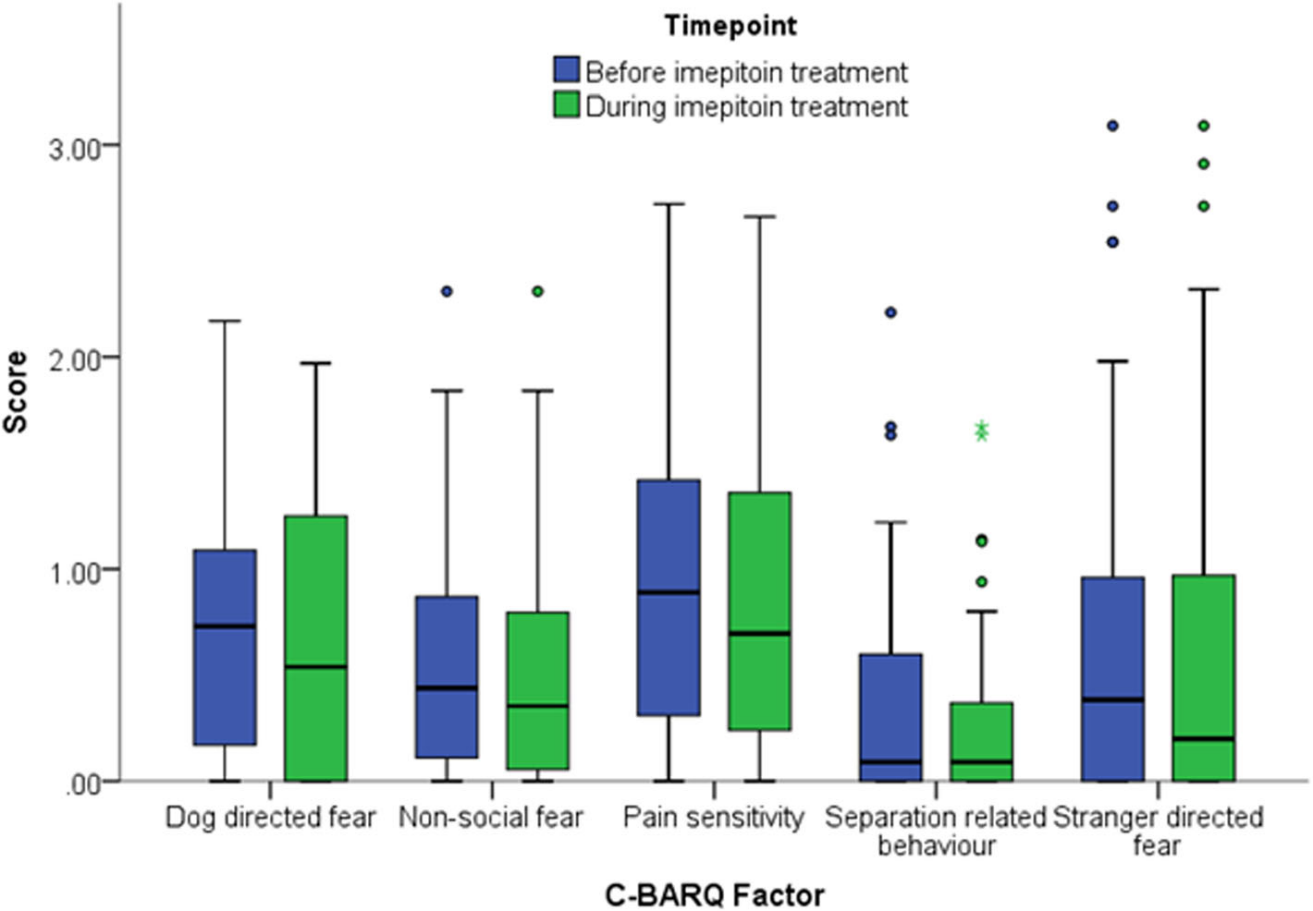
Box and whisker plot of the five C-BARQ behavioural measures before and after imepitoin treatment in dogs in dogs treated with only imepitoin as a monotherapy (*n* = 56). The top and bottom lines of the boxes represent the 25th and 75th percentiles, the central line indicates the median, and the whiskers represent the range. Filled circles represent outliers and stars represent extreme outliers

No significant effect of imepitoin treatment was found on the five behavioural measures, in either the whole population (Table 1) or when only monotherapy cases were considered (*p* > 0.05) (Fig. 2).

**Table 1:** Five C-BARQ behavioural measures before and after imepitoin treatment in dogs treated with imepitoin with or without other anti-epileptic drugs

Behavioural measure	Before imepitoin treatment	During imepitoin treatment	Wilcoxon (W)	N	p
Dog directed fear	0.40 (0–1.09)	0.54 (0–1.09)	265.5	83	0.660
Stranger directed fear	0.19 (0–0.81)	0.19 (0–0.99)	206.0	84	0.946
Non-social fear	0.36 (0.09–0.78)	0.38 (0.11–0.85)	505.0	84	0.108
Pain sensitivity	0.62 (0–1.29)	0.62 (0.24–1.33)	307.0	83	0.125
Separation related behaviour	0.0 (0–0.33)	0.09 (0–0.47)	268	80	0.058

Data are presented as the median accompanied by 25th–75th percentiles

When only those monotherapy cases that were drug-naive (had received no AED treatment prior to imepitoin treatment, *n* = 43) were analysed, there was still no difference observed in any of the five behavioural measures before and during treatment (*p* > 0.05). As dogs were not selected based on high anxiety levels, to investigate the effect of imepitoin on only those dogs with higher levels of anxiety within this population, analyses were repeated including only those dogs with CBARQ scores above the population median for each factor (Dog directed fear: 0.543, Stranger directed fear: 0.203, Non-social fear: 0.351, Pain sensitivity: 0.680, Separation related behaviour: 0.088). Again, no effect of imepitoin treatment upon the five measures was found in this sub-population (*p* > 0.05).

There was no effect of signalment (sex, neuter status or breed) on any of the five behavioural measures before or during imepitoin treatment (*p* > 0.05). There was no correlation between changes in any of the five behavioural measures before or during imepitoin treatment and drug response (as quantified by percentage change (%) in seizure frequency; *p* > 0.05), or with the side effects scores observed in either the first 2 weeks, or after 2 weeks of treatment (*p* > 0.05).

### Impact on seizure frequency

Significant reductions in seizures/month and clusters/month were reported by owners while their dog was treated with imepitoin compared to before imepitoin treatment; however no significant change was seen in the number of seizure days/month (Table 2). The median reduction in seizure frequency following imepitoin treatment (number of seizures/month) was −1 (−2 to 0), and the median percentage change in seizure frequency was a 45% reduction (−89.6% - 0%) across all cases.

**Table 2:** Changes in seizure frequency following imepitoin treatment in both monotherapy and polytherapy cases

Seizure frequency measure	Before imepitoin treatment	During imepitoin treatment	Wilcoxon (W)	N	P
Seizures/month	3.11 ± 4.02	1.93 ± 2.86	1256.0	76	0.002
Seizure days/month	2.31 ± 3.47	1.92 ± 3.30	802.5	74	0.105
Cluster seizure episodes/ month	0.91 ± 1.23	0.65 ± 1.34	461.0	77	0.038

Data are presented as the median accompanied by 25th–75th percentiles

There was no significant difference in % reduction in seizure frequency between dogs with or without cluster seizures (Clusters median: 0% (−73.2% – 0%) vs. single seizures median: −58.3% (−100% - 0%); Mann-Whitney U = 863, *p* = 0.073). There was no significant difference in % reduction in seizure frequency between dogs being treated with imepitoin as a monotherapy or polytherapy (Monotherapy median: −50% (−100% - 0%) vs. polytherapy median: 0% (−76.3%- +100%); Mann-Whitney *U* = 820.5, *p* = 0.058). There was no association between the number of seizures before initiation of imepitoin treatment and the percentage change (%) in seizure frequency (KW = 3.11, *p* = 0.375), or any effect of sex, neuter status or breed on percentage change (%) in seizure frequency (*p* > 0.05).

### Side effects profile

The most common and severe side effect reported in both the first 2 weeks and after 2 weeks of imepitoin treatment was polyphagia. There was a significant reduction in the side effect score before and after 2 weeks of treatment, with the median side effect score in the first 2 weeks 13 out of 55 (4–19.5) and after the initial 2 weeks 9 out of 55 (1.5–15.5) (W = 247.5, *p* < 0.001) (Table 3).

**Table 3:** Side effect score (out of 5) in the first 2 weeks and after 2 weeks of treatment with imepitoin in dogs treated with imepitoin with or without other anti-epileptic drugs

Side effect	All cases (*n* = 85) (Median (25th–75th percentile)		Just imepitoin monotherapy cases (*n* = 56) (Median (25th–75th percentile)	
	Initial 2 weeks	After 2 weeks	Initial 2 weeks	After 2 weeks
Eating more / would like to eat more	3 (0–4)	2 (0–4)	2 (0–4)	1 (0–4)
Gaining weight	0 (0–3)	0 (0–2)	0 (0–3)	0 (0–2)
Drinking more	2 (0–3)	1 (0–3)	1 (0–3)	1 (0–2.75)
Urinating more	1 (0–3)	0 (0–2.5)	0 (0–3)	0 (0–2)
Sleeping more than before	1 (0–3)	0 (0–2)	1 (0–3)	0 (0–2.75)
Wobbly / not coordinated when walking	0 (0–3)	0 (0–1)	0 (0–1.75)	0 (0–0)
Restlessness / pacing	0 (0–3)	0 (0–2)	0 (0–2)	0 (0–1)
Itchiness or skin rash	0 (0–0)	0 (0–0)	0 (0–0)	0 (0–0)
Vomiting	0 (0–0)	0 (0–0)	0 (0–0)	0 (0–0)
Diarrhoea	0 (0–0)	0 (0–0)	0 (0–0)	0 (0–0)
Coughing	0 (0–0)	0 (0–0)	0 (0–0)	0 (0–0)
OVERALL SCORE (out of 55)	13 (4–19.5)	9 (1.5–15.5)	10 (2–16)	7 (1–13.75)

Side effects were rated by owners on a six point scale from side effect not present (0), very mild (1), mild (2), moderate (3), severe (4), and very severe (5)

The side effect score was greater in polytherapy cases than in monotherapy cases in both the initial 2 weeks (median monotherapy 10 (2–16) vs. median polytherapy 18 (8–24.5); Mann-Whitney U = 1098.5, *p* = 0.008) and after the initial 2 weeks (median monotherapy 7 (1–13.75) vs. median polytherapy 12 (7–18); Mann-Whitney *U* = 1044.5, *p* = 0.030). In the first 2 weeks, differences in side effect frequencies between monotherapy and polytherapy cases were observed for: increased urinating (*X* ^2^ = 18.1, *p* = 0.03) and ataxia (*X* ^2^ = 12.7, *p* = 0.026) which were more common in polytherapy cases. After 2 weeks, differences in side effect frequencies between monotherapy and polytherapy cases were observed for: increased appetite (*X* ^2^ = 11.5, *p* = 0.04), increased urinating (*X* ^2^ = 11.4, *p* = 0.04), increased sleeping (*X* ^2^ = 11.6, *p* = 0.04) and ataxia (*X* ^2^ = 14.7, *p* = 0.01) which were all more common in polytherapy cases.

There was no correlation between treatment efficacy (percentage change (%) in seizure frequency) or the seizure frequency before imepitoin treatment was initiated and the side effect score in either period (*p* < 0.05). There was no relationship between side effect score and any aspect of signalment (*p* > 0.05).

## Discussion

This study for the first time investigated the impact of an anti-epileptic drug used to treat seizures on fear and anxiety-related behaviour in dogs with IE. The results indicated that none of the five fear and anxiety-related behavioural measures changed significantly following treatment with imepitoin. It is possible that despite being a partial GABA_A_ agonist, imepitoin does not have the same anxiolytic effects as other BDZ receptor agonists or partial agonists, or that this effect does not occur in dogs with IE. In line with previous reports, anti-epileptic effects were seen in the majority of dogs, with a median reduction in seizure frequency of 45%. Imepitoin side effects were generally mild and short-lived, with a significant reduction in the number and severity of side effects observed after the initial 2 weeks of treatment. Ataxia, a common AED side effect [30] was mild or not present in the majority of cases, in line with recent objective evidence demonstrating reduced levels in IE patients treated with imepitoin compared to phenobarbitone [31]. A greater number of side effects were seen in dogs treated with imepitoin alongside other AEDs, which is in line with recent studies; for example, when imepitoin was used as an add-on therapy to phenobarbital, or vice versa, the number of side effects increased [32]. In addition, a recent study of imepitoin polytherapy found that 69% of dogs treated with imepitoin and KBr, and 79% of dogs treated with imepitoin and phenobarbital experienced side effects [33].

The lack of behavioural effects seen in this study may be due to the IE study population not exhibiting a severe enough initial anxiety level to allow for a significant effect to be seen. Investigating the effects of imepitoin upon the behaviour of dogs with recognised behavioural anxiety-related problems (e.g. specific fears and phobias, separation-related behaviours) +/− behavioural management steps (e.g. desensitisation and counter-conditioning [34]) is required to further explore any potential anxiolytic effects of this medication in general. This may extend beyond dogs, with imepitoin recently tested in clinically healthy cats in a randomised, controlled, blinded study, and found to be well tolerated [35]. In a case study of a 15 year old Persian cat with night-time vocalisation, 8 months of imepitoin treatment was found to control the screaming which may indicate anxiolytic effects [36]. Further placebo-controlled studies on a larger scale are required to confirm this effect in both species.

Studying the anxiolytic effects of imepitoin in healthy dogs and cats without epilepsy may provide further insights into imepitoin’s efficacy as a behavioural medicine, due to the potential differences between the healthy and epileptic brain. It is possible that imepitoin may be an effective anxiolytic in the ‘normal’ brain, but ineffective in the epileptic brain (particularly in drug-resistant patients). The majority of studies of the anxiolytic effects of imepitoin have used either healthy animals or induced models of epilepsy. Imepitoin has shown mixed results in tests of anxiety in experimental animals (rats and mice) [15, 37–45]. In rats, although anxiolytic activity was shown in the elevated plus maze, Vogel conflict test, light-dark chamber tests and social interaction test, these effects were not seen in the Geller conflict test, a model in which prior food-deprived rats have to choose between consuming food and avoiding the punishment associated with this consumption. In mice, anxiolytic activity was shown in the elevated plus maze and light-dark chamber tests, but not in the four-plate test, where mice are punished upon crossing the border between four plates.

It is possible that behavioural changes such as anxiety may be a sign of the natural progression of IE that becomes worse over time. Imepitoin may have reduced the progression of these changes in the current study (hence the lack of change in the measured behavioural traits); however, without a control group of dogs with IE that were not treated with imepitoin or other AEDs over a comparable period, this could not be detected in the current study. Understanding the progression of epilepsy and its behavioural comorbidities is of high priority in interpreting the effects of treatment interventions for both seizure activity and behaviour.

Dog owners were used as the raters of canine behaviour in this study, as online questionnaires allow for rapid acquisition of data on a sufficient number of animals for statistical purposes. Despite this positive aspect of the study methodology, it is possible that owners may not always be reliable raters of canine behaviour [46]. Questions in several of the studied C-BARQ measures are prefixed with “*Please assess the following situations and determine how likely your dog is to respond in a fearful or anxious way*”, and it is possible that owners were unable to correctly ascertain which behaviours would indicate their dog was fearful or anxious in a situation. This may not have had a marked effect on the current study, as fear is one of the most easily recognised emotional states by owners in dogs [46, 47]. Including objective measures of fear and anxiety alongside owner reports may clarify the results of future studies. The success of existing anxiolytics is often limited to owner reports of anxiety reduction after a period of treatment. Herron et al. (2008) evaluated the effects of diazepam in dogs with anxiety-related behaviour problems; however, their evaluation of efficacy was based on owners rating the effectiveness of diazepam for its prescribed indication as ‘very effective’, ‘moderately effective’, ‘slightly effective’, or ‘not effective’ [24]. Although a convenient way to question an owner’s perception of treatment success, results cannot be accurately compared among owners, as perception of efficacy may vary between individuals.

As these data were collected retrospectively, recall bias may have reduced the reliability of the responses received. Recall time varied between owners, with a median of 263.9 days (106.3–478.4 days) since imepitoin treatment commenced. Although this relatively long time on this treatment allows sufficient time for effects to be observed, it may be challenging for owners to accurately recall their dog’s behaviour before imepitoin treatment was initiated. To improve upon this limitation, future studies should prospectively study the effects of imepitoin and other AEDs on canine behaviour in a longitudinal manner.

Despite the apparent lack of anxiolytic effects in this study population, the anti-epileptic properties of imepitoin were apparent. A median 45% reduction in owner-reported seizure frequency/month was observed following imepitoin treatment. Significant reductions in seizure frequency were observed following imepitoin treatment for the measures ‘number of seizures/month’ and ‘number of clusters/month’; however, no difference was found in number of seizure days/month. This may indicate that the overall number of seizures that occurred was reduced within each day (i.e. clusters), but the number of days they occurred upon did not change. The effectiveness of imepitoin to treat dogs with cluster seizures has not been studied to date, and thus imepitoin is not recommended as a primary treatment for dogs with cluster seizures [48]. Over half of the dogs studied (55.3%, *n* = 47) had experienced cluster seizures in this study despite this recommendation, with no changes in treatment efficacy found between clustering and non-clustering dogs. As this study was not designed to assess the efficacy of imepitoin as a treatment for cluster seizures, further prospective studies are required to investigate whether this is an appropriate treatment option for this sub-population of dogs with epilepsy.

Seizure freedom remains the holy grail of epilepsy treatment, and 14.1% of dogs became seizure free during imepitoin treatment which is in line with some existing estimates of AED-related remission [49]. However, the remission rate observed in this study was significantly lower than a previous investigation of imepitoin efficacy, where complete suppression of generalized seizures was observed in 46.9% of dogs (30/64) [19]. Remission rates may differ significantly depending on study design, duration of follow up and epilepsy phenotype of the patients studied, and thus the dogs in this study may have exhibited a more severe, drug-resistant form of IE. The collection of retrospective seizure frequency data from owners is a limitation of this study, as data may be liable to recall bias and dependent upon the reliability of the owner’s record keeping. The use of electronic seizure diaries, which are becoming increasingly used by owners, is likely to improve the reliability of owner records, which are vital to epilepsy studies involving client-owned animals. Encouraging owners to reliably record seizures, using the same format of seizure diaries may improve comparability of results across studies in the future.

Polytherapy is commonly used in the management of canine epilepsy, and when imepitoin monotherapy and polytherapy cases were considered separately, 17.9% of monotherapy cases became seizure free in comparison to only 6.9% of polytherapy cases. This is unsurprising, as it has previously been demonstrated that drug response diminishes with the successive use of AEDs [50]. No predictors were found that were significantly associated with imepitoin drug-response and future research should focus on this to elucidate why some dogs do not experience a reduction in seizure frequency, or experience an increase in seizure frequency when receiving imepitoin, while others become seizure free. Understanding whether using imepitoin in polytherapy with other AEDs leads to significant improvements in seizure frequency is of importance, as greater side effects were present in polytherapy patients in this study. Managing the balance of seizure control vs. the impact of side effects remains a challenge in the optimisation of quality of life in epilepsy patients [4, 51]. Exploring alternative management options, including dietary therapy, may be of benefit in the management of seizure activity and anxiety in dogs experiencing adverse effects of polytherapy. Supplementation of alpha-casozepine and L-tryptophan [52] and a diet containing *Valeriana officinalis*, *Melissa officinalis* and tryptophan [53] have been reported to reduce anxiety-related behaviour in dogs; however, neither has been studied in dogs with epilepsy. In a recent randomised, placebo-controlled, double-blinded crossover trial of a ketogenic diet containing 10% medium chain triglycerides, a reduction in stranger-directed fear was observed alongside reduced seizure frequency in dogs with idiopathic epilepsy, which may indicate anxiolytic effects [54].

## Conclusions

The data from this study are unable to provide evidence of the anxiolytic effects of imepitoin when primarily used to treat idiopathic epilepsy in dogs; however, anti-epileptic effects were seen in the majority of dogs with generally mild and short-lived side effects. It is possible that anxiety levels in the IE population studied were not sufficiently high to demonstrate an anxiety-reducing effect of imepitoin, and thus targeting dogs with or without epilepsy with recognised behavioural problems may allow anxiolytic effects to be detected in future prospective studies.

## Abbreviations

AED: Anti-epileptic drug
BDZ: Benzodiazepine
C-BARQ: Canine behavioural assessment and research questionnaire
GABA: Gamma-aminobutyric acid
IE: Idiopathic epilepsy
KBr: Potassium bromide
LEV: Levetiracetam
MRI: Magnetic resonance image
PB: Phenobarbital
QoL: Quality of life
SE: Standard error
SSRI: Selective serotonin reuptake inhibitor
TCA: tri-cyclic anti-depressant
